# Serine-Aspartate Repeat Protein D Increases Staphylococcus aureus Virulence and Survival in Blood

**DOI:** 10.1128/IAI.00559-16

**Published:** 2016-12-29

**Authors:** Fatemeh Askarian, Satoshi Uchiyama, J. Andrés Valderrama, Clement Ajayi, Johanna U. E. Sollid, Nina M. van Sorge, Victor Nizet, Jos A. G. van Strijp, Mona Johannessen

**Affiliations:** a Research Group of Host-Microbe Interactions, Department of Medical Biology, Faculty of Health Sciences, UiT, The Arctic University of Norway, Tromsø, Norway; b Department of Pediatrics and Skaggs School of Pharmacy and Pharmaceutical Sciences, University of California, San Diego, California, USA; c Department of Medical Microbiology, University Medical Center Utrecht, Utrecht, The Netherlands; University of Texas at Austin

**Keywords:** SdrD, virulence, neutrophils, systemic infection, whole blood

## Abstract

Staphylococcus aureus expresses a panel of cell wall-anchored adhesins, including proteins belonging to the microbial surface components recognizing adhesive matrix molecule (MSCRAMM) family, exemplified by the serine-aspartate repeat protein D (SdrD), which serve key roles in colonization and infection. Deletion of *sdrD* from S. aureus subsp. aureus strain NCTC8325-4 attenuated bacterial survival in human whole blood *ex vivo*, which was associated with increased killing by human neutrophils. Remarkably, SdrD was able to inhibit innate immune-mediated bacterial killing independently of other S. aureus proteins, since addition of recombinant SdrD protein and heterologous expression of SdrD in Lactococcus lactis promoted bacterial survival in human blood. SdrD contributes to bacterial virulence *in vivo*, since fewer S. aureus subsp. aureus NCTC8325-4 Δ*sdrD* bacteria than bacteria of the parent strain were recovered from blood and several organs using a murine intravenous infection model. Collectively, our findings reveal a new property of SdrD as an important key contributor to S. aureus survival and the ability to escape the innate immune system in blood.

## INTRODUCTION

Staphylococcus aureus is a human commensal that persistently colonizes the anterior nares of healthy individuals but is also a leading cause of multiple community-acquired and nosocomial infections ranging from mild skin and soft tissue infections to life-threatening invasive diseases, including sepsis and endocarditis (1). The bacterium expresses various cell wall-anchored proteins, including the microbial surface components recognizing adhesive matrix molecules (MSCRAMMs). Among the MSCRAMMs are the clumping factor (Clf)–serine-aspartate repeat (Sdr) group of proteins, including SdrD (2). SdrD promotes the adherence of S. aureus to desquamated nasal epithelial cells (3) and to human keratinocytes *in vitro* (4) and contributes to abscess formation *in vivo* (5).

Neutrophils, the most abundant circulating phagocytes of the innate immune system, play an important role in protection against pathogen dissemination (6, 7). Decoration of bacteria with opsonins, i.e., specific immunoglobulins and components of the complement system, activates the phagocytic machinery through interaction with the corresponding neutrophil Fc receptors or complement receptors (reviewed in reference 8). The generation of reactive oxygen species (ROS) through activation of NADPH oxidase and expression of antibacterial molecules in granules is a key mechanism for neutrophil-mediated killing of phagocytized bacteria, while elaboration of DNA-based neutrophil extracellular traps (NETs) promotes extracellular microbial killing (reviewed in reference 8). A crucial role of neutrophils in controlling S. aureus infection is evident through the study of patients with immune defects (reviewed in reference 9); for example, cancer patients with neutropenia (10) or individuals with genetic defects of neutrophil function (11, 12) demonstrate markedly increased rates of S. aureus infection.

S. aureus can enter the bloodstream through several routes (1), whereupon a sophisticated and synergistic group of neutrophil resistance mechanisms, including antioxidant systems, factors that bind or inactivate granule components, and mechanisms to avoid complement opsonization and phagocytosis, enhance the *in vivo* fitness of the organism (6, 13, 14). Impairment of neutrophil intracellular killing allows S. aureus to survive long enough within these cells in the bloodstream to travel to and infect distant sites (reviewed in references 6 and 15).

A higher prevalence of the *sdrD* gene among S. aureus isolates from patients with bone infections has been reported (16, 17). In addition, the expression of *sdrD* is upregulated upon incubation in fresh human blood *ex vivo* (18). These data raised the possibility that SdrD could play a role during systemic infection. To date the interaction of SdrD with host innate immune components has not been studied. The current work evaluates whether SdrD may aid the pathogen in immune evasion. Using an isogenic mutant with a deletion of the *sdrD* gene, we found a significant contribution of SdrD in S. aureus resistance to killing by innate immune components present in blood *in vitro* and bacterial clearance from tissues and blood during systemic infections.

## RESULTS

### Expression profile of *sdrD*.

**FIG 1:**
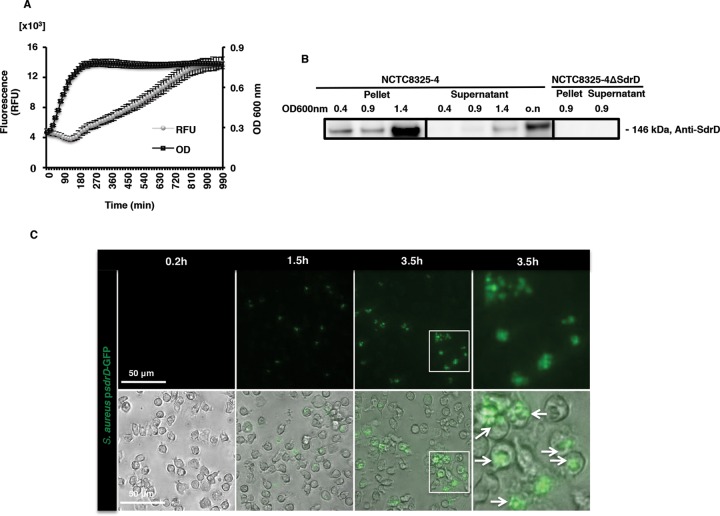
*sdrD* promoter activity and SdrD protein expression under different growth conditions. (A) Promoter activity of *sdrD* during growth in TSB using NCTC8325-4 harboring the *sdrD*-GFP reporter construct (S. aureus subsp. aureus p*sdrD*-GFP). Data represent the means ± SEMs from an individual experiment. The experiments were performed twice in triplicate. RFU, relative fluorescence units. (B) Immunoblotting of the bacterial lysates and the culture cell-free supernatant of NCTC8325-4 and its isogenic mutant, NCTC8325-4 Δ*sdrD*, using anti-SdrD antibody on the bacterial lysates and the culture cell-free supernatant. A representative Western blot is shown. (C) Promoter activity of *sdrD* evaluated by fluorescence microscopy using NCTC8325-4 harboring p*sdrD*-GFP in the presence of freshly isolated human neutrophils. (Top) Live imaging was performed after 0.2, 1.5, and 3.5 h using fluorescence microscopy; (bottom) bright-field and GFP merged images. The white boxes in the first column labeled 3.5 h are enlarged in the second column labeled 3.5 h. The experiment was performed at an MOI of 20. Arrows, S. aureus subsp. aureus p*sdrD*-GFP. The sizes of the scale bars are indicated.

The expression profile of *sdrD* was assessed using S. aureus subsp. aureus NCTC8325-4 expressing an *sdrD* promoter-green fluorescent protein (GFP) fusion construct. In regular bacterial growth medium (tryptic soy broth [TSB]), the level of *sdrD* promoter-driven GFP expression was low and slightly decreased during early exponential growth. Thereafter, the *sdrD* promoter activity increased when bacteria entered late exponential phase and continued to increase during the stationary phase (Fig. 1A).

We also tested SdrD protein expression by immunoblotting using SdrD-specific antibodies. SdrD was detected within the bacterial lysate of NCTC8325-4 and was detected most abundantly at early stationary phase but was not detected in the isogenic mutant, NCTC8325-4 Δ*sdrD* (Fig. 1B). Similar to a previous report for SdrC (19), SdrD was detected in cell-free concentrated culture supernatants, particularly in late exponential phase (Fig. 1B), which may have been due to proteolytic release and/or cell death.

A previous study showed that *sdrD* expression was upregulated in the presence of human whole blood (18). As neutrophils are the most abundant leukocytes in blood, we examined *sdrD* promoter activity in the presence of freshly isolated human neutrophils. *sdrD* promoter-driven expression of GFP in NCTC8325-4 occurred after 1.5 and 3.5 h upon coincubation with neutrophils *in vitro* at multiplicities of infection (MOI) of 20 (Fig. 1C) and 10 (results not shown). *sdrD* promoter activity was independent of S. aureus extracellular or intracellular localization (Fig. 1C, 3.5 h).

### SdrD promotes S. aureus survival in blood.

**FIG 2:**
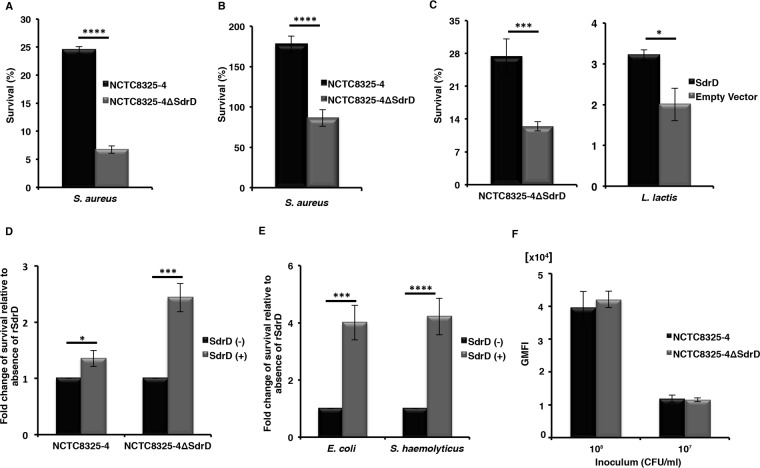
SdrD promotes S. aureus survival in human and mouse whole blood. (A and B) SdrD increases the rate of survival of NCTC8325-4 in human blood (A) and in mouse blood (B). The rate of survival for bacteria inoculated at time zero was arbitrarily set equal to 100%, and the rate of survival after 3 h is presented as a percentage of the number of bacteria in the inoculum. (C) Transcomplementation of NCTC8325-4 Δ*sdrD* with plasmid-expressed SdrD (left) and ectopic expression of SdrD in L. lactis (right) restore survival in human whole blood. Bacterial survival was analyzed by viability counting. The rate of survival is presented as described in the legend to panel A. (D) Influence of rSdrD (12 μg/ml) on the survival of NCTC8325-4 and NCTC8325-4 Δ*sdrD* in human whole blood. The rate of survival in untreated blood was arbitrarily set equal to 1, and the rate of bacterial survival in blood treated with rSdrD is represented as the fold change. (E) The survival of E. coli and S. haemolyticus in human whole blood is increased in the presence of rSdrD (12 μg/ml). The fold change in bacterial survival is presented as described in the legend to panel D. (F) NCTC8325-4 and its mutant, NCTC8325-4 Δ*sdrD*, were labeled with FITC, and 40 μl of different concentrations of inoculum (∼1 × 10^8^ or 1 × 10^7^ CFU/ml) was incubated with human whole blood. Data represent the geometric mean of the fluorescence intensity (GMFI). Data are presented as the means ± SEMs from at least three independent experiments. Statistical analysis was performed by Student's *t* test. Significant differences are indicated by asterisks. *, *P* < 0.05; ***, *P* ≤ 0.001; ****, *P* ≤ 0.0001.

Since *sdrD* promoter activity increased in the presence of neutrophils (Fig. 1C) and in the presence of blood (18), we hypothesized that SdrD may affect bacterial survival in blood. To address this hypothesis, NCTC8325-4 and NCTC8325-4 Δ*sdrD* were incubated in 80% freshly drawn blood, using hirudin as an anticoagulant. After 3 h of incubation in human blood, 25% of the SdrD-expressing inoculum survived, while only 7% of the isogenic mutant was recovered (*P* < 0.05) (Fig. 2A). The approximately 3.5-fold higher rate of survival of the parent strain was not attributable to differences in bacterial growth rates between NCTC8325-4 and its isogenic mutant (see Fig. S1A in the supplemental material) (4). The SdrD-mediated increase in bacterial survival was also observed in mouse blood (Fig. 2B).

To corroborate the specificity of SdrD-mediated bacterial survival in blood, a blood survival assay was performed using the complementation and heterologous expression systems in NCTC8325-4 Δ*sdrD* and Lactococcus lactis. As seen in Fig. 2C, NCTC8325-4 Δ*sdrD* and L. lactis ectopically expressing SdrD survived better in human blood than L. lactis carrying only the empty vector (Fig. 2C, left and right, respectively). Moreover, addition of purified, recombinant full-length SdrD (rSdrD) increased the rates of survival of NCTC8325-4 and its isogenic mutant in human whole blood 1.3- and 2.4-fold, respectively (Fig. 2D). Finally, a blood survival assay was performed using Staphylococcus haemolyticus and Escherichia coli in the absence or presence of exogenously administered rSdrD. The presence of recombinant SdrD strongly promoted the survival of both strains in human whole blood (Fig. 2E), underscoring the ability of SdrD to protect the pathogen from immune killing.

In summary, these lines of evidence demonstrate that the presence of SdrD improves the survival of bacteria in human and mouse blood.

### The presence of SdrD does not affect S. aureus phagocytosis *ex vivo*.

To assess whether the SdrD-mediated increase in survival in blood was due to variation in bacterial uptake by neutrophils, we tested its contribution in a human whole-blood phagocytosis assay. Fluorescence-labeled NCTC8325-4 and NCTC8325-4 Δ*sdrD* were incubated with 80% human whole blood. Neutrophil-mediated phagocytosis was analyzed by flow cytometry. We observed that the levels of uptake of NCTC8325-4 and NCTC8325-4 Δ*sdrD* by human neutrophils were comparable (Fig. 2F). Similar results were obtained when 25 and 50% human whole blood was used and rSdrD was adding to the assay mixture (Fig. S2A and B). Thus, the SdrD-mediated increased survival in human whole blood is not explained by differences in phagocytosis.

### SdrD attenuates neutrophil-mediated S. aureus killing.

**FIG 3:**
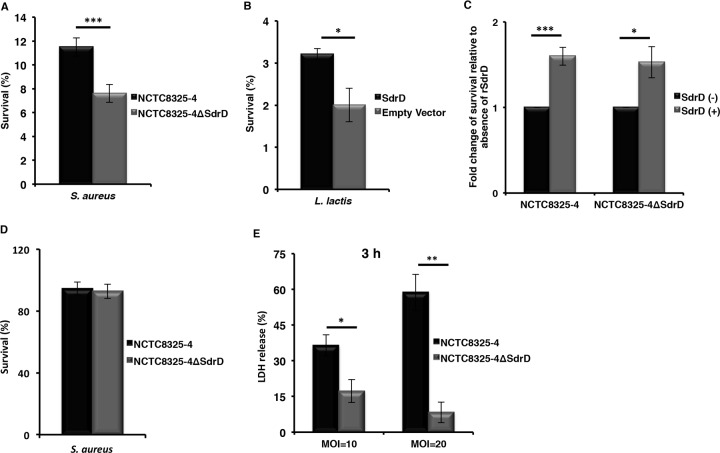
SdrD promotes S. aureus survival in the presence of human neutrophils. (A and B) Survival of NCTC8325-4 and NCTC8325-4 Δ*sdrD* (A) and L. lactis containing *sdrD*-pMG36e or the empty vector (B) after incubation with freshly isolated human neutrophils. The bacteria were opsonized with plasma and exposed to purified neutrophils before bacterial survival was analyzed by viability counting. The rate of survival for the bacterial inoculum was arbitrarily set equal to 100%, and the rate of survival of the bacteria after 1 h is presented as a percentage of the number of bacteria in the inoculum. (C) Influence of rSdrD on survival of plasma-opsonized NCTC8325-4 or NCTC48325-4 Δ*sdrD* in the presence of human neutrophils. Bacterial survival was analyzed by viability counting. The survival of untreated neutrophils was arbitrarily set equal to 1, and the rate of bacterial survival in the neutrophils treated with rSdrD is presented as the fold change. (D) PMA-induced neutrophils were used to perform a NET-mediated killing assay using NCTC8325-4 or NCTC48325-4 Δ*sdrD* at an MOI of 1. The bacteria that were incubated without neutrophils served as controls, and the rate of survival for these bacteria was arbitrarily set equal to 100%. The rate of survival of these bacteria after 30 min is presented as a percentage of the number of bacteria in the inoculum. (E) Freshly isolated neutrophils were exposed to NCTC8325-4 or NCTC48325-4 Δ*sdrD* at an MOI of either 10 or 20. Percent viability was calculated by measuring the amount of LDH released from the cytosol of damaged cells into the supernatant. Data represents means ± SEMs from at least three independent experiments. Statistical analysis was performed by Student's *t* test. Significant differences are indicated by asterisks. *, *P* < 0.05; **, *P* ≤ 0.01; ***, *P* ≤ 0.001.

To further probe how SdrD may contribute to bacterial survival in human blood, the role of SdrD in S. aureus survival in the presence of neutrophils was investigated. For this purpose, plasma-opsonized S. aureus subsp. aureus (NCTC8325-4 and its isogenic mutant) and L. lactis (with or without an expression plasmid carrying SdrD) were coincubated with neutrophils for 1 h in the presence of human plasma and plated for determination of the number of surviving bacteria. Expression of SdrD significantly increased the rate of survival of the parent strain (Fig. 3A) compared to that of the isogenic mutant. The increased survival was not due to differences in bacterial growth in RPMI 1640 supplemented with human serum albumin (HSA), Todd-Hewitt broth (THB), or plasma-HSA (Fig. S1A and B). Expression of SdrD also mediated enhanced survival of L. lactis in the presence of neutrophils (Fig. 3B). We then tested whether exogenously added SdrD promoted the survival of S. aureus in the presence of neutrophils and found that addition of rSdrD increased the rates of survival of both NCTC8325-4 and NCTC8325-4 Δ*sdrD* (Fig. 3C). These results reveal that SdrD can contribute to neutrophil resistance.

### S. aureus expressing SdrD does not show increased survival in NETs but influences neutrophil viability.

The role of NETs in the extracellular killing of pathogens has been previously demonstrated (reviewed in reference 20). To determine whether SdrD protected S. aureus against NET-mediated extracellular killing, neutrophils were treated with phorbol 12-myristate 13-acetate (PMA) for 4 h to induce maximal NET production before addition of bacteria. The rates of survival of NCTC8325-4 and NCTC8325-4 Δ*sdrD* were comparable after 30 min of exposure to NETs (Fig. 3D). The same results were obtained using opsonized bacteria (results not shown). These results suggest that SdrD does not contribute to S. aureus survival in NETs.

Next, we asked whether resistance to neutrophil killing influenced neutrophil viability. Freshly isolated human neutrophils were infected with NCTC8325-4 and NCTC8325-4 Δ*sdrD* at MOIs of 10 and 20. Cell viability was monitored by measuring the amount of intracellular lactate dehydrogenase (LDH) released after 1 and 3 h. The percentage of LDH released was less than 10% and independent of SdrD expression after 1 h of infection at MOIs of 10 and 20 (results not shown). After 3 h of coincubation, NCTC8325-4 induced a significantly higher level of LDH release from neutrophils than NCTC8325-4 Δ*sdrD* (Fig. 3E), indicating that the higher rate of survival of SdrD-containing bacteria in the presence of neutrophils may promote or, indeed, in part, reflect pathogen-induced cell death.

### SdrD promotes S. aureus survival in blood and multiple organs in a murine intravenous infection model.

**FIG 4:**
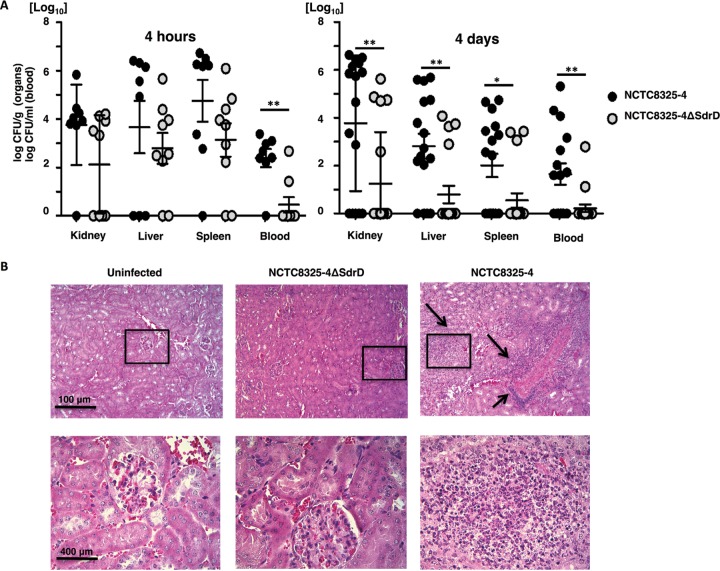
SdrD increases the bacterial burden in a murine systemic infection model. (A) The bacterial loads in the kidney, liver, spleen, and blood (number of CFU per gram for organs and number of CFU per milliliter for blood) of 8-week-old female CD-1 mice were enumerated 4 h and 4 days after systemic infection with NCTC8325-4 and NCTC8325-4 Δ*sdrD*. The data represent the means ± SEMs from an individual experiment for data obtained at 4 h postinfection and two independent experiments for data obtained at 4 days postinfection. Statistical analysis was performed by Student's *t* test. Significant differences are indicated by asterisks. *, *P* < 0.05; **, *P* ≤ 0.01. (B) Thin sections of H&E-stained uninfected kidney tissue and kidney tissue infected with S. aureus subsp. aureus NCTC8325-4 and NCTC8325-4 Δ*sdrD* collected on day 4 postinfection were analyzed by light microscopy. Arrows, infiltrate of neutrophils. The sizes of the scale bars are indicated.

Finally, we evaluated whether SdrD impacted S. aureus virulence *in vivo*. CD-1 mice were injected intravenously with NCTC8325-4 or NCTC8325-4 Δ*sdrD*. After 4 h and 4 days, organs and blood were harvested and the numbers of CFU were determined. Deletion of *sdrD* resulted in significantly decreased S. aureus loads in the blood at 4 h postinfection (Fig. 4A, left), while the levels of recovery of NCTC8325-4 and NCTC8325-4 Δ*sdrD* from kidney, spleen, and liver were comparable. At 4 days postinfection, a significantly higher level of recovery of NCTC8325-4 than NCTC8325-4 Δ*sdrD* was apparent in blood as well as in spleen, kidney, and liver (Fig. 4A, right).

Histopathological evaluation of kidney as a target organ for S. aureus in a bacteremia model of infection (21) was performed to determine the extent of NCTC8325-4-induced organ damage. Tissues from uninfected mice and NCTC8325-4 Δ*sdrD*-infected mice were included for comparison (Fig. 4B). Examination of the kidneys at 4 days after intravenous inoculation of S. aureus revealed increased neutrophil infiltration in 2/6 examined mice inoculated with NCTC8325-4 (Fig. 4B, right), while increased neutrophil infiltration was not observed in the kidneys of mice infected with NCTC8325-4 Δ*sdrD* (Fig. 4B, middle).

Collectively, these data suggest the contribution of SdrD to S. aureus virulence *in vivo*.

## DISCUSSION

Several of the MSCRAMM proteins are multifunctional and play a role in adhesion, invasion, and/or immune evasion (2). MSCRAMMs play a significant role in life-threatening infections, such as sepsis, pneumonia, and endocarditis, as well as prosthetic device infections (2). Indeed, studies in mice have revealed that sortase A S. aureus mutants are less virulent than wild-type S. aureus strains in bloodstream infections (22) and less able to form abscesses in mice (22, 23). In addition, ClfA, ClfB, IsdA, IsdB, and SdrD were also found to contribute to the general process of infection *in vivo* (5).

Approximately 15% of S. aureus genes encode proven or putative virulence factors (24). We hypothesized that SdrD may be a virulence factor since *sdrD* is upregulated in the presence of human whole blood (19). Neutrophils are the most abundant immune cell type in blood, and here we found induction of *sdrD*-driven GFP expression over time in the presence of neutrophils independently of phagocytic uptake of bacteria (Fig. 1C). Upon exposure to human plasma, bacterial engulfment by neutrophils is strongly promoted by opsonization of the microbe with either complement activation products at the C3 level (C3b, iC3b) or antibodies (25–27). The interference of certain MSCRAMMs, such as ClfA, SdrE, and collagen adhesin (Cna), with complement-mediated opsonization and bacterial killing by neutrophils has been previously demonstrated (28–32). An *ex vivo* human whole-blood assay was performed using NCTC8325-4 and *sdrD* mutant (NCTC8325-4 Δ*sdrD*) strains in the presence of staphylococcal complement inhibitor (SCIN), which blocks the activity of the C3 convertases on the bacterial surface (27). In contrast to ClfA and SdrE, SdrD increases bacterial survival in whole blood independently of impaired C3b deposition (results not shown) and phagocytosis (Fig. 2F and S2).

SdrD provides a survival advantage to opsonized bacteria in the presence of neutrophils (Fig. 3A to C). The intracellular survival of S. aureus within neutrophils has been previously demonstrated (33–35), and spontaneous microbial escape through host cell lysis may ensue (33, 34), contributing to systemic infection *in vivo* (35). Resistance to killing and survival inside neutrophils are ways for S. aureus to subvert innate immune responses (34; reviewed in reference 7). Moreover, resistance to killing can contribute to bacterial spread by allowing the phagocytic cell to serve as a transport vehicle (reviewed in references 6, 8, and 15). The SdrD-mediated increase in the rate of bacterial survival in blood, resistance to neutrophil killing, and spread in the host may indeed be consequences of host cell lysis, since SdrD-expressing NCTC8325-4 caused increased neutrophil lysis after 3 h of incubation *in vitro* (Fig. 3E). One mechanism of SdrD contributing to S. aureus virulence thus appears to be the counteracting of neutrophil defenses, which thereby enhances bacterial spread in the host. In agreement with this, a significantly higher bacterial load of S. aureus subsp. aureus NCTC8325-4 was found in multiple organs of infected mice at 4 days postinfection than in isogenic mutant-infected mice (Fig. 4A, right). However, the exact mechanism by which SdrD increases bacterial survival and influences the viability of neutrophils remains elusive.

SdrD-expressing S. aureus bacteria showed increased survival *in vivo* (Fig. 4A), which suggests a role of SdrD in S. aureus resistance to immune clearance and systemic disease pathogenesis. Infection with S. aureus subsp. aureus NCTC8325-4 resulted in local neutrophil accumulation in the kidney tissues (Fig. 4B). Several MSCRAMMs, including SdrD, have previously been found to be required for S. aureus abscess formation, where bacteria are protected against host immunity through pseudocapsule formation in a neutrophil-rich site (5). The molecular mechanism for this remains elusive, but SdrD is assumed to be involved at stage II, where neutrophils, eventually with surviving intracellular S. aureus, attract a massive infiltrate of immune cells, including additional neutrophils (5). Moreover, exposure of the parent strain to pooled human plasma prior to the neutrophil killing assay increased NCTC8325-4 resistance to clearance by immune cells. S. aureus expresses multiple virulence factors, e.g., clumping factor A (ClfA) and clumping factor B (ClfB) (36) and bone sialoprotein-binding protein (BbP) (37), that utilize plasma coagulation proteins to protect the bacteria from immune defense and promote bacterial proliferation and persistence. However, whether SdrD specifically attracts clotting factors from the plasma to the bacterial surface and whether this is a prerequisite for abscess formation as a requirement for S. aureus persistence in host tissues remain to be determined.

There is considerable functional redundancy among staphylococcal virulence factors in subverting their host defense targets. Thus, identification of individual virulence factors in a well-armed clinical isolate poses various challenges. We took advantage of the laboratory strain NCTC8325-4, which does not express SdrE (38), is capsule negative (see references 39 and 40 and references therein), and has a defect in the expression of ClfA (41, 42). The use of such a strain facilitates the identification of a whole-blood survival phenotype conferred by SdrD, since functional redundancy is less prominent. Our results were confirmed by use of a surrogate strain expressing SdrD (Fig. 2C, right) and clearly add credence to the mechanistic role of SdrD in immune evasion. Whether the immune-evasive function of SdrD contributes to pathogenesis in clinical isolates has yet to be established. However, the presence of MSCRAMMs varies among strains (38), and as all S. aureus clones have the potential to cause invasive infections under the right circumstances (37, 38), SdrD may be of special importance for strains lacking certain other immune-evasive surface proteins.

In summary, using *in vitro* and *in vivo* models of systemic infection, we found that SdrD increased the rate of S. aureus survival, which may result in increased bacterial fitness in the host. A definitive ligand for SdrD in human whole blood remains elusive, and further research is merited to probe the precise molecular mechanisms underlying SdrD-mediated immune evasion by a well-armed clinical S. aureus isolate.

## MATERIALS AND METHODS

### Bacterial strains and growth conditions.

S. aureus subsp. aureus NCTC8325-4, S. aureus subsp. aureus NCTC8325-4 Δ*sdrD*, Lactococcus lactis MG1363 expressing SdrD (*sdrD*-pMG36e), and L. lactis MG1363 expressing the empty vector (pMG36e) have been described previously (4). Moreover, the *sdrD*-pMG36e and pMG36e constructs (4) were used to complement NCTC8325-4 Δ*sdrD* through electroporation (100-Ω resistance, 25-μF capacitance, and 2.5-kV voltage). E. coli ATCC 25922 (43) and Staphylococcus haemolyticus 51-30 (a clinical isolate recovered from blood; Rikshospitalet, Oslo, Norway) were also included.

Overnight cultures of S. aureus or L. lactis were diluted 1:100 in tryptic soy broth (TSB; Sigma-Aldrich, Germany) or SMG17 (M17 broth supplemented with 0.5 M sucrose [Sigma-Aldrich, Germany] and 0.5% glucose [Sigma-Aldrich, Germany]) and incubated at 37°C and 220 rpm or 30°C without shaking, respectively. S. haemolyticus and E. coli were grown in TSB or brain heart infusion (BHI), respectively. Bacterial growth was monitored by determination of the optical density at 600 nm (OD_600_). Bacteria were harvested at an OD_600_ of 0.8, pelleted by centrifugation, washed twice in phosphate-buffered saline (PBS; Biochrom, Germany), and diluted to the selected number of CFU per milliliter or OD_600_ in RPMI 1640 (Gibco, Life Technologies, UK) containing 0.05% human serum albumin (HSA; Sanquin, Amsterdam, The Netherlands) or 2% (vol/vol) fetal bovine serum (FBS; Invitrogen Life Technologies, USA), depending on the experimental setting.

### *sdrD* gene expression using S. aureus subsp. aureus p*sdrD*-GFP.

Expression of *sdrD* in S. aureus subsp. aureus NCTC8325-4 was evaluated using a reporter construct. The S. aureus subsp. aureus *sdrD*-GFP reporter strain was described previously (4) and was subsequently used to assess *sdrD* expression during growth in TSB medium. An overnight culture of S. aureus subsp. aureus NCTC8325-4 harboring the *sdrD* reporter construct (S. aureus subsp. aureus p*sdrD*-GFP) was diluted 1:100 in prewarmed TSB medium, incubated at 37°C under shaking conditions, and harvested at an OD_600_ of 0.4. The bacteria were pelleted, washed in PBS, and diluted in TSB medium. Fluorescence was measured under shaking conditions using a Synergy H1 hybrid reader (BioTek, USA) with excitation and emission of 488 and 520 nm, respectively. A control sample of S. aureus subsp. aureus NCTC8325-4 without the GFP reporter construct was included for background correction.

### SdrD protein expression profile.

Expression of the SdrD protein was assessed by immunoblot analysis. Lysates of S. aureus subsp. aureus NCTC8325-4 and the isogenic NCTC8325-4 Δ*sdrD* mutant were prepared for Western blotting as described previously (4). To assess the release of SdrD, the culture supernatant was filter sterilized (pore size, 0.22 μm) to remove intact bacterial cells and concentrated using an Amicon Ultra 50K centrifugal filter device (Millipore Corp., USA). Expression of SdrD in the bacterial pellet and supernatant was evaluated by immunoblotting using SdrD A region-specific (a kind gift from Elisabet Josefsson) (primary) and polyclonal swine anti-rabbit immunoglobulin (Dako, Denmark) (secondary) antibodies.

### Fluorescence microscopy analysis of *sdrD* expression upon coculture with neutrophils.

Expression of *sdrD* in S. aureus subsp. aureus NCTC8325-4 in the presence of freshly isolated neutrophils was evaluated using an *sdrD* promoter-GFP reporter construct as described previously (44). Neutrophils were freshly isolated from heparinized venous blood of healthy volunteers using Histopaque (Sigma-Aldrich, Germany)-Ficoll (GE Healthcare, Sweden) or 1-step Polymorphprep solution (Fresenius Kabi Norge AS, Norway) gradient centrifugation. S. aureus subsp. aureus p*sdrD*-GFP was prepared as described above in “*sdrD* gene expression using S. aureus subsp. aureus p*sdrD*-GFP” with minor modifications (44). Neutrophils (2 × 10^5^) in RPMI 1640 (no phenol red)–FBS were added to a 96-well plate (Corning, USA). Bacteria were added to the neutrophils at a multiplicity of infection (MOI) of 10 or 20. The plate was incubated at 37°C in a CO_2_ incubator (5% CO_2_). Representative live images were collected by a Zeiss AxioObserver D1 microscope (Zeiss, Germany) using both bright-field and GFP fluorescence at times of 0.2, 1.5, and 4 h.

### Blood survival assay.

Blood samples from healthy volunteers or CD-1 mice were collected in tubes containing hirudin (Roche, Switzerland) as the anticoagulant to preserve complement activity. One hundred sixty microliters of freshly drawn blood was mixed with 20 μl volumes containing S. aureus (∼1 × 10^7^ CFU/ml), S. haemolyticus (∼1 × 10^6^ CFU/ml), or E. coli (∼1 × 10^7^ CFU/ml) or 40 μl of L. lactis (∼1 × 10^8^ CFU/ml) in RPMI 1640–HSA. When applicable, 20 μl of RPMI 1640–HSA or recombinant full-length SdrD (rSdrD) (4) in RPMI 1640–HSA was added to the samples in siliconized tubes (Sigma-Aldrich, Germany) at a final concentration of 12 μg/ml. After incubation for 3 h at 37°C on a horizontal rotator, the blood cells were lysed by addition of 1 ml ice-cold H_2_O supplemented with 0.3% saponin (Sigma-Aldrich, Germany). Bacterial survival was evaluated by serial dilution on blood or Todd-Hewitt broth (THB) agar plates. Percent survival was determined by comparing the number of surviving bacteria to the number of bacteria in the inoculum.

### Growth curves for S. aureus subsp. aureus NCTC8325-4 and NCTC8325-4 Δ*sdrD*.

S. aureus subsp. aureus NCTC8325-4 and NCTC8325-4 Δ*sdrD* were grown overnight in THB. On the next day, the bacteria were washed twice in PBS, resuspended in RPMI 1640–HSA or RPMI 1640 supplemented with 5% bacteriologic medium (THB), and added to 96-well plates (Corning, USA) in a total volume of 200 μl. Growth was monitored by measuring the OD_600_ every 15 min for 24 h under shaking conditions using a Bioscreen CMBR machine (Growth Curves USA). Bacterial growth in RPMI 1640–HSA in the presence of plasma was also quantified using serial dilutions on THB agar plates.

### Whole-blood phagocytosis.

For fluorescent labeling, bacteria were resuspended in PBS containing 0.5 mg/ml fluorescein isothiocyanate (FITC; Sigma-Aldrich, Germany) for 20 to 30 min on ice and protected from light. Bacteria were washed extensively in PBS and then resuspended in RPMI 1640–HSA to an OD_600_ of 0.4. Forty microliters of FITC-labeled S. aureus subsp. aureus NCTC8325-4 and NCTC8325-4 Δ*sdrD* (∼1 × 10^8^ or 1 × 10^7^ CFU/ml) was incubated for 15 to 30 min at 37°C with freshly isolated human blood (80%, 50%, and 25% blood in RPMI 1640–HSA) anticoagulated with hirudin. The reaction was stopped using fluorescence-activated cell sorter lysing solution (BD Biosciences, USA). Samples were washed with PBS supplemented with 1% bovine serum albumin (BSA; Sigma-Aldrich, Germany) and analyzed by flow cytometry. When indicated, rSdrD at a final concentration of 3 μg/ml was added to the samples. Gating of cells was carried out on the basis of forward and side scatter. The fluorescence intensity (FL) of 10,000 gated neutrophils was measured for each sample using a flow cytometer (BD Biosciences, USA). Phagocytosis was identified when neutrophils expressed fluorescence. The geometric mean of the fluorescence intensity (GMFI) was calculated using FlowJo software.

### Neutrophil killing assay.

Bacteria were opsonized with hirudin-anticoagulated plasma and incubated with freshly isolated neutrophils at an MOI of 10 for S. aureus or an MOI of 20 for L. lactis in siliconized tubes. rSdrD was added to a final concentration of 12 μg/ml when indicated. Samples were incubated at 37°C with vigorous shaking. After 1 h, 1 ml of ice-cold H_2_O supplemented with 0.3% saponin was added, and the mixture was incubated on ice for 5 min. Surviving bacteria were quantified by serial dilution on THB agar plates. Percent survival was determined by comparing the numbers of surviving bacteria to the number of inoculated bacteria.

### Neutrophil viability assays.

Neutrophil viability in the presence of S. aureus subsp. aureus NCTC8325-4 and NCTC8325-4 Δ*sdrD* was measured through LDH release (Promega, USA). Briefly, neutrophils (2 × 10^5^ cells/ml) in RPMI 1640–HSA were seeded into a 96-well plate. Bacteria were added to the wells at an MOI of 10 or 20. The culture supernatants were collected after 1 and 3 h, and cytotoxicity was measured on a multimode plate reader (EnSpire Alpha; PerkinElmer, USA) according to the manufacturer's instructions.

### NET-mediated killing assay.

The NET-mediated killing assay was performed as described previously (45) with minor modifications. Freshly isolated neutrophils (2 × 10^6^ cells/ml) in RPMI 1640–FBS were seeded in 12-well plates and stimulated with 25 nM phorbol 12-myristate 13-acetate (PMA; Sigma-Aldrich, Germany) for 4 h at 37°C to induce NET release. Bacteria were added at an MOI of 1, followed by centrifugation onto the NETs (500 × *g*, 5 min), and then incubated at 37°C for 30 min. Wells containing bacteria without neutrophils served as a control. The content of each well was scraped thoroughly and pipetted vigorously. Surviving bacteria were quantified by serial dilution on THB agar plates. Percent survival was determined by comparing the numbers of surviving bacteria in the presence of NETs to the numbers in the control wells.

### Murine model of intravenous infection.

An established model of S. aureus systemic infection (46) was used to determine the difference in virulence between S. aureus subsp. aureus NCTC8325-4 and the isogenic NCTC8325-4 Δ*sdrD* mutant. Eight-week-old female CD-1 mice (Charles River, Wilmington, MA, USA) were infected intravenously with approximately 9 × 10^6^ CFU of NCTC8325-4 or NCTC8325-4 Δ*sdrD* bacteria by tail vein injection. The bacterial load (given as the number of CFU per gram of organ tissue or the number of CFU per milliliter of blood) in the kidney, spleen, liver, and blood was quantified at 4 h and 4 days postinfection by homogenizing the organs and plating serial dilutions on THP agar plates. Data represent the means ± standard errors of the means (SEMs) of data from an individual experiment for 4 h postinfection (*n* = 8 for NCTC8325-4 and *n* = 9 for NCTC8325-4 Δ*sdrD*) and the means ± SEMs of data pooled from two independent experiments for 4 days postinfection (*n* = 8 for NCTC8325-4 and *n* = 9 for NCTC8325-4 Δ*sdrD* per experiment).

### Histopathology analysis.

The kidneys of the infected animals (*n* = 6 for NCTC8325-4 and *n* = 6 for NCTC8325-4 Δ*sdrD*) were dissected and fixed in 10% paraformaldehyde (PFA) for 24 h (2 ml of PFA/100 mg of tissue) and later moved into 70% ethanol for long-term storage until the tissues were embedded in paraffin wax. Kidney sections (5 μm thick) were deparaffinized through immersion in xylene (3 times for 10 min each time) and rehydrated by passing them back through decreasing concentrations of ethanol (100 and 95%, 2 times for 5 min each time). The sections were stained with hematoxylin and eosin (H&E) and evaluated microscopically using an Olympus BX51 microscope (Olympus Europe GmbH, Germany).

### Ethical approval.

Human blood analysis was carried out in accordance with the ethical principles of the Helsinki Declaration, the medical ethics committee of the University Medical Center Utrecht (Utrecht, The Netherlands), and ethical approval 2014/1653 REK North-Norway. Mouse studies were performed under approved protocol S00227M of the University of California, San Diego, Institutional Animal Care and Use Committee.

### Statistical analysis.

Statistical analysis of the pooled data from the experiments was carried out. Student's *t* test in Excel software (Fig. 1 to 3) and GraphPad Prism software (Fig. 4) was used for determination of statistically significant differences between groups (*P* < 0.05). Excel software (Fig. 1 to 3 and the figures in the supplemental material) and GraphPad Prism software (Fig. 4) were used to generate the graphs.

## Supplementary Material

Supplemental material

## References

[1] Malachowa N, DeLeo FR 2011 *Staphylococcus aureus* survival in human blood. Virulence 2:567–569. doi:10.4161/viru.2.6.17732. 21971187 PMC3260549

[2] Foster TJ, Geoghegan JA, Ganesh VK, Hook M 2014 Adhesion, invasion and evasion: the many functions of the surface proteins of *Staphylococcus aureus*. Nat Rev Microbiol 12:49–62. doi:10.1038/nrmicro3161. 24336184 PMC5708296

[3] Corrigan RM, Miajlovic H, Foster TJ 2009 Surface proteins that promote adherence of *Staphylococcus aureus* to human desquamated nasal epithelial cells. BMC Microbiol 9:22. doi:10.1186/1471-2180-9-22. 19183486 PMC2642834

[4] Askarian F, Ajayi C, Hanssen AM, van Sorge NM, Pettersen I, Diep DB, Sollid JUE, Johannessen M 2016 The interaction between Staphylococcus aureus SdrD and desmoglein 1 is important for adhesion to host cells. Sci Rep 6:22134. doi:10.1038/srep22134. 26924733 PMC4770587

[5] Cheng AG, Kim HK, Burts ML, Krausz T, Schneewind O, Missiakas DM 2009 Genetic requirements for *Staphylococcus aureus* abscess formation and persistence in host tissues. FASEB J 23:3393–3404. doi:10.1096/fj.09-135467. 19525403 PMC2747682

[6] Spaan AN, Surewaard BG, Nijland R, van Strijp JA 2013 Neutrophils versus *Staphylococcus aureus*: a biological tug of war. Annu Rev Microbiol 67:629–650. doi:10.1146/annurev-micro-092412-155746. 23834243

[7] Anwar S, Prince LR, Foster SJ, Whyte MK, Sabroe I 2009 The rise and rise of Staphylococcus aureus: laughing in the face of granulocytes. Clin Exp Immunol 157:216–224. doi:10.1111/j.1365-2249.2009.03950.x. 19604261 PMC2730847

[8] van Kessel KP, Bestebroer J, van Strijp JA 2014 Neutrophil-mediated phagocytosis of *Staphylococcus aureus*. Front Immunol 5:467. doi:10.3389/fimmu.2014.00467. 25309547 PMC4176147

[9] Thammavongsa V, Kim HK, Missiakas D, Schneewind O 2015 Staphylococcal manipulation of host immune responses. Nat Rev Microbiol 13:529–543. doi:10.1038/nrmicro3521. 26272408 PMC4625792

[10] Chemaly RF, Hachem RY, Husni RN, Bahna B, Abou Rjaili G, Waked A, Graviss L, Nebiyou Bekele B, Shah JN, Raad II 2010 Characteristics and outcomes of methicillin-resistant Staphylococcus aureus surgical-site infections in patients with cancer: a case-control study. Ann Surg Oncol 17:1499–1506. doi:10.1245/s10434-010-0923-5. 20127184

[11] Spickett GP 2008 Immune deficiency disorders involving neutrophils. J Clin Pathol 61:1001–1005. doi:10.1136/jcp.2007.051185. 18755725

[12] Lakshman R, Finn A 2001 Neutrophil disorders and their management. J Clin Pathol 54:7–19. doi:10.1136/jcp.54.1.7. 11271792 PMC1731272

[13] Foster TJ 2005 Immune evasion by staphylococci. Nat Rev Microbiol 3:948–958. doi:10.1038/nrmicro1289. 16322743

[14] Nizet V 2007 Understanding how leading bacterial pathogens subvert innate immunity to reveal novel therapeutic targets. J Allergy Clin Immunol 120:13–22. doi:10.1016/j.jaci.2007.06.005. 17606031

[15] Thwaites GE, Gant V 2011 Are bloodstream leukocytes Trojan horses for the metastasis of *Staphylococcus aureus*? Nat Rev Microbiol 9:215–222. doi:10.1038/nrmicro2508. 21297670

[16] Trad S, Allignet J, Frangeul L, Davi M, Vergassola M, Couve E, Morvan A, Kechrid A, Buchrieser C, Glaser P, El-Solh N 2004 DNA macroarray for identification and typing of *Staphylococcus aureus* isolates. J Clin Microbiol 42:2054–2064. doi:10.1128/JCM.42.5.2054-2064.2004. 15131170 PMC404631

[17] Sabat A, Melles DC, Martirosian G, Grundmann H, van Belkum A, Hryniewicz W 2006 Distribution of the serine-aspartate repeat protein-encoding sdr genes among nasal-carriage and invasive *Staphylococcus aureus* strains. J Clin Microbiol 44:1135–1138. doi:10.1128/JCM.44.3.1135-1138.2006. 16517913 PMC1393123

[18] Sitkiewicz I, Babiak I, Hryniewicz W 2011 Characterization of transcription within sdr region of *Staphylococcus aureus*. Antonie Van Leeuwenhoek 99:409–416. doi:10.1007/s10482-010-9476-7. 20571861 PMC3032192

[19] Barbu EM, Ganesh VK, Gurusiddappa S, Mackenzie RC, Foster TJ, Sudhof TC, Hook M 2010 beta-Neurexin is a ligand for the *Staphylococcus aureus* MSCRAMM SdrC. PLoS Pathog 6:e1000726. doi:10.1371/journal.ppat.1000726. 20090838 PMC2800189

[20] von Kockritz-Blickwede M, Blodkamp S, Nizet V 2016 Interaction of bacterial exotoxins with neutrophil extracellular traps: impact for the infected host. Front Microbiol 7:402. doi:10.3389/fmicb.2016.00402. 27064864 PMC4811905

[21] von Kockritz-Blickwede M, Rohde M, Oehmcke S, Miller LS, Cheung AL, Herwald H, Foster S, Medina E 2008 Immunological mechanisms underlying the genetic predisposition to severe Staphylococcus aureus infection in the mouse model. Am J Pathol 173:1657–1668. doi:10.2353/ajpath.2008.080337. 18974303 PMC2626378

[22] Rauch S, Gough P, Kim HK, Schneewind O, Missiakas D 2014 Vaccine protection of leukopenic mice against *Staphylococcus aureus* bloodstream infection. Infect Immun 82:4889–4898. doi:10.1128/IAI.02328-14. 25183728 PMC4249340

[23] Mazmanian S, Liu G, Jensen E, Lenoy E, Schneewind O 2000 *Staphylococcus aureus* sortase mutants defective in the display of surface proteins and in the pathogenesis of animal infections. Proc Natl Acad Sci U S A 97:5510–5515. doi:10.1073/pnas.080520697. 10805806 PMC25859

[24] Malachowa N, Whitney AR, Kobayashi SD, Sturdevant DE, Kennedy AD, Braughton KR, Shabb DW, Diep BA, Chambers HF, Otto M, DeLeo FR 2011 Global changes in *Staphylococcus aureus* gene expression in human blood. PLoS One 6:e18617. doi:10.1371/journal.pone.0018617. 21525981 PMC3078114

[25] Rooijakkers SH, van Kessel KP, van Strijp JA 2005 Staphylococcal innate immune evasion. Trends Microbiol 13:596–601. doi:10.1016/j.tim.2005.10.002. 16242332

[26] Gasque P 2004 Complement: a unique innate immune sensor for danger signals. Mol Immunol 41:1089–1098. doi:10.1016/j.molimm.2004.06.011. 15476920

[27] Rooijakkers SH, Ruyken M, Roos A, Daha MR, Presanis JS, Sim RB, van Wamel WJ, van Kessel KP, van Strijp JA 2005 Immune evasion by a staphylococcal complement inhibitor that acts on C3 convertases. Nat Immunol 6:920–927. doi:10.1038/ni1235. 16086019

[28] Hair PS, Echague CG, Sholl AM, Watkins JA, Geoghegan JA, Foster TJ, Cunnion KM 2010 Clumping factor A interaction with complement factor I increases C3b cleavage on the bacterial surface of *Staphylococcus aureus* and decreases complement-mediated phagocytosis. Infect Immun 78:1717–1727. doi:10.1128/IAI.01065-09. 20100856 PMC2849425

[29] Hair P, Foster T, Cunnion K 2008 Clumping factor A increases complement factor I binding to the *Staphylococcus aureus* surface and increases surface iC3b generation. Mol Immunol 45:4165.

[30] Sharp JA, Echague CG, Hair PS, Ward MD, Nyalwidhe JO, Geoghegan JA, Foster TJ, Cunnion KM 2012 *Staphylococcus aureus* surface protein SdrE binds complement regulator factor H as an immune evasion tactic. PLoS One 7:e38407. doi:10.1371/journal.pone.0038407. 22675461 PMC3364985

[31] Kang M, Ko YP, Liang X, Ross CL, Liu Q, Murray BE, Hook M 2013 Collagen-binding microbial surface components recognizing adhesive matrix molecule (MSCRAMM) of Gram-positive bacteria inhibit complement activation via the classical pathway. J Biol Chem 288:20520–20531. doi:10.1074/jbc.M113.454462. 23720782 PMC3711317

[32] Hair PS, Wagner SM, Friederich PT, Drake RR, Nyalwidhe JO, Cunnion KM 2012 Complement regulator C4BP binds to Staphylococcus aureus and decreases opsonization. Mol Immunol 50:253–261. doi:10.1016/j.molimm.2012.01.010. 22333221

[33] Voyich JM, Braughton KR, Sturdevant DE, Whitney AR, Said-Salim B, Porcella SF, Long RD, Dorward DW, Gardner DJ, Kreiswirth BN, Musser JM, DeLeo FR 2005 Insights into mechanisms used by *Staphylococcus aureus* to avoid destruction by human neutrophils. J Immunol 175:3907–3919. doi:10.4049/jimmunol.175.6.3907. 16148137

[34] Greenlee-Wacker MC, Rigby KM, Kobayashi SD, Porter AR, DeLeo FR, Nauseef WM 2014 Phagocytosis of Staphylococcus aureus by human neutrophils prevents macrophage efferocytosis and induces programmed necrosis. J Immunol 192:4709–4717. doi:10.4049/jimmunol.1302692. 24729616 PMC4011196

[35] Gresham HD, Lowrance JH, Caver TE, Wilson BS, Cheung AL, Lindberg FP 2000 Survival of *Staphylococcus aureus* inside neutrophils contributes to infection. J Immunol 164:3713–3722. doi:10.4049/jimmunol.164.7.3713. 10725730

[36] Deivanayagam CC, Perkins S, Danthuluri S, Owens RT, Bice T, Nanavathy T, Foster TJ, Hook M, Narayana SV 1999 Crystallization of ClfA and ClfB fragments: the fibrinogen-binding surface proteins of *Staphylococcus aureus*. Acta Crystallogr D Biol Crystallogr 55:554–556. doi:10.1107/S0907444998012426. 10089377

[37] Vazquez V, Liang X, Horndahl JK, Ganesh VK, Smeds E, Foster TJ, Hook M 2011 Fibrinogen is a ligand for the *Staphylococcus aureus* microbial surface components recognizing adhesive matrix molecules (MSCRAMM) bone sialoprotein-binding protein (Bbp). J Biol Chem 286:29797–29805. doi:10.1074/jbc.M110.214981. 21642438 PMC3191021

[38] McCarthy AJ, Lindsay JA 2010 Genetic variation in Staphylococcus aureus surface and immune evasion genes is lineage associated: implications for vaccine design and host-pathogen interactions. BMC Microbiol 10:173. doi:10.1186/1471-2180-10-173. 20550675 PMC2905362

[39] Wann ER, Dassy B, Fournier JM, Foster TJ 1999 Genetic analysis of the cap5 locus of Staphylococcus aureus. FEMS Microbiol Lett 170:97–103. doi:10.1111/j.1574-6968.1999.tb13360.x. 9919657

[40] Kuipers A, Stapels DA, Weerwind LT, Ko YP, Ruyken M, Lee JC, van Kessel KP, Rooijakkers SH 2016 The Staphylococcus aureus polysaccharide capsule and Efb-dependent fibrinogen shield act in concert to protect against phagocytosis. Microbiology 162:1185–1194. doi:10.1099/mic.0.000293. 27112346 PMC4977062

[41] O'Neill AJ 2010 Staphylococcus aureus SH1000 and 8325-4: comparative genome sequences of key laboratory strains in staphylococcal research. Lett Appl Microbiol 51:358–361. doi:10.1111/j.1472-765X.2010.02885.x. 20618890

[42] Entenza JM, Moreillon P, Senn MM, Kormanec J, Dunman PM, Berger-Bachi B, Projan S, Bischoff M 2005 Role of sigmaB in the expression of Staphylococcus aureus cell wall adhesins ClfA and FnbA and contribution to infectivity in a rat model of experimental endocarditis. Infect Immun 73:990–998. doi:10.1128/IAI.73.2.990-998.2005. 15664942 PMC547034

[43] Minogue TD, Daligault HA, Davenport KW, Bishop-Lilly KA, Broomall SM, Bruce DC, Chain PS, Chertkov O, Coyne SR, Freitas T, Frey KG, Gibbons HS, Jaissle J, Redden CL, Rosenzweig CN, Xu Y, Johnson SL 2014 Complete genome assembly of Escherichia coli ATCC 25922, a serotype O6 reference strain. Genome Announc 2(5):e00969-14. doi:10.1128/genomeA.00969-14. 25291776 PMC4175212

[44] Surewaard BG, van Strijp JA, Nijland R 2013 Studying interactions of Staphylococcus aureus with neutrophils by flow cytometry and time lapse microscopy. J Vis Exp 2013:e50788. doi:10.3791/50788. PMC3814803 23893048

[45] Parker H, Albrett AM, Kettle AJ, Winterbourn CC 2012 Myeloperoxidase associated with neutrophil extracellular traps is active and mediates bacterial killing in the presence of hydrogen peroxide. J Leukoc Biol 91:369–376. doi:10.1189/jlb.0711387. 22131345

[46] Askarian F, van Sorge NM, Sangvik M, Beasley FC, Henriksen JR, Sollid JU, van Strijp JA, Nizet V, Johannessen M 2014 A Staphylococcus aureus TIR domain protein virulence factor blocks TLR2-mediated NF-kappaB signaling. J Innate Immun 6:485–498. doi:10.1159/000357618. 24481289 PMC4198549

