# Supplementary material for: Serine-Aspartate Repeat Protein D Increases Staphylococcus aureus Virulence and Survival in Blood
Source: Infect Immun. 2016 Dec 29;85(1):e00559-16. doi: 10.1128/IAI.00559-16 (PMC5203653; doi:10.1128/IAI.00559-16)
Supplement: Supplemental material [file supp_85_1_e00559-16__index.html]

Supplemental material 

# Serine-Aspartate Repeat Protein D Increases Staphylococcus aureus Virulence and Survival in Blood

## Supplemental material

- Supplemental file 1 -

  Fig. S1. *S. aureus* growth under different experimental conditions.

  PDF, 123K
- Supplemental file 2 -

  Fig. S2. SdrD does not affect whole-blood phagocytosis.

  PDF, 75K
- Supplemental file 3 -

  Fig. S1 and S2 legends.

  PDF, 77K
