# Supplementary figures and images for: Serine-Aspartate Repeat Protein D Increases Staphylococcus aureus Virulence and Survival in Blood
Source: Infect Immun. 2016 Dec 29;85(1):e00559-16. doi: 10.1128/IAI.00559-16 (PMC5203653; doi:10.1128/IAI.00559-16)

**A**

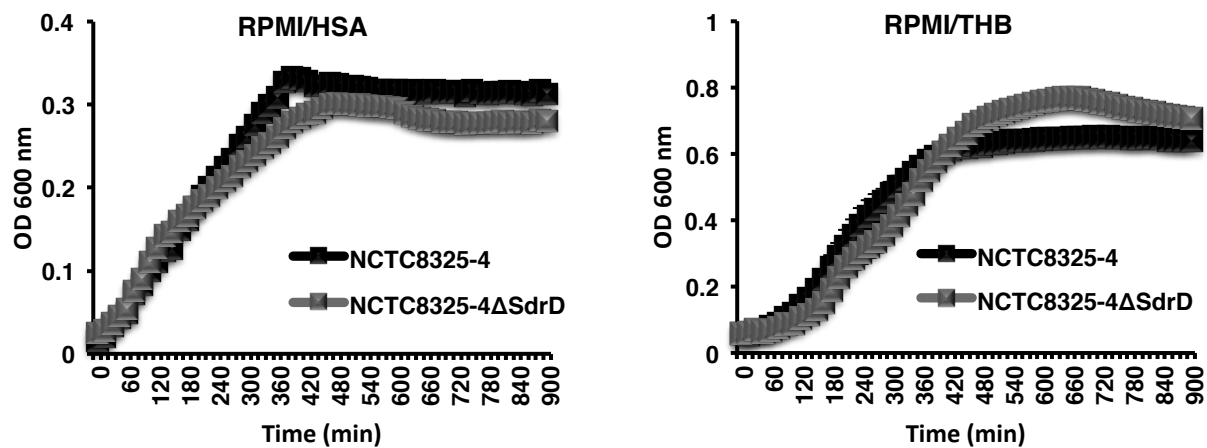

**B**

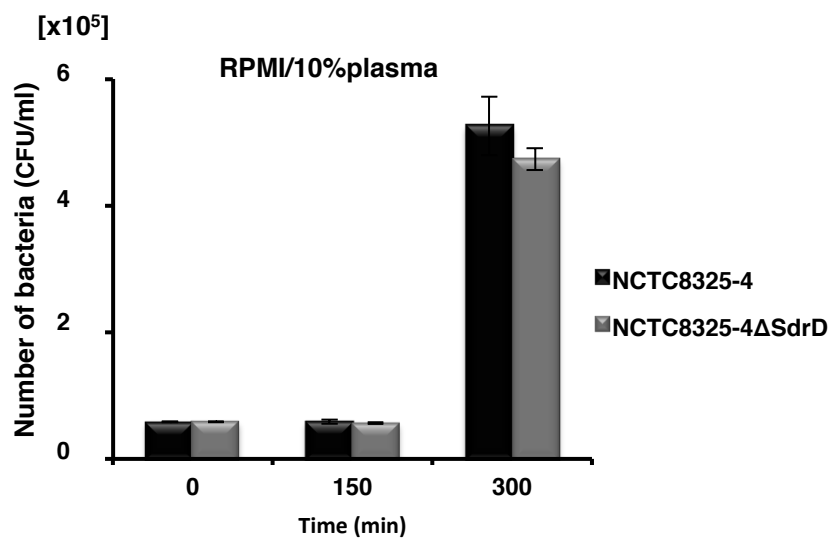

Supplement: Supplemental material [file IAI.00559-16_zii999091922s1.pdf]

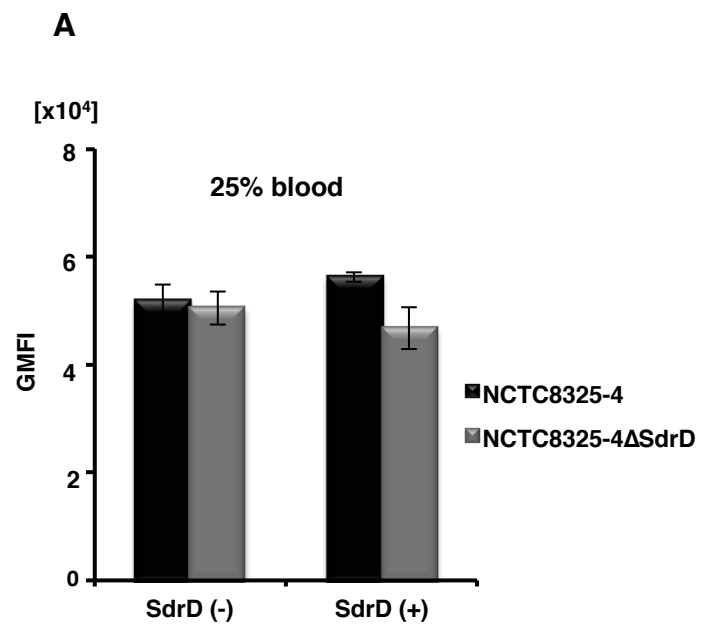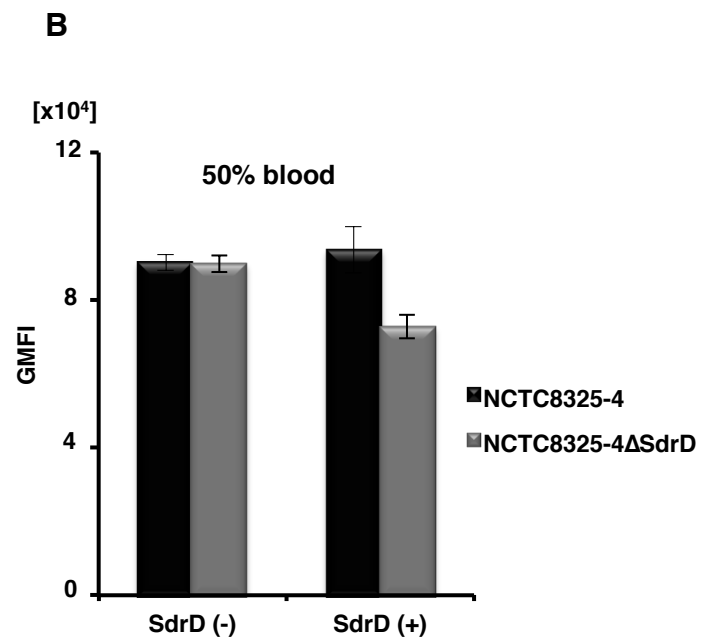

Supplement: Supplemental material [file IAI.00559-16_zii999091922s2.pdf]
