# Supplementary material for: Serine-Aspartate Repeat Protein D Increases Staphylococcus aureus Virulence and Survival in Blood
Source: Infect Immun. 2016 Dec 29;85(1):e00559-16. doi: 10.1128/IAI.00559-16 (PMC5203653; doi:10.1128/IAI.00559-16)
Supplement: Supplemental material [file IAI.00559-16_zii999091922s3.pdf]

Supplementary Figure 1. ***S. aureus*** **growth in different experimental conditions. (A)** Growth curves of S. aureus NCTC8325-4 and its isogenic mutant NCTC8325-4ΔsdrD in RPMI/HSA and RPMI/THB. Data represents means ± SEM of three independent experiments. **(B)** NCTC8325-4 and NCTC8325-4ΔsdrD were inoculated in RPMI/HSA containing 10% pooled hirudin plasma. Bacterial numbers were enumerated at different time points by serial dilution plating. Data represents means ± SEM of an individual experiment performed in triplicate.

Supplementary Figure 2. SdrD does not affect whole blood phagocytosis. *S. aureus* NCTC8325-4 and its mutants NCTC8325-4Δ*sdrD* were labeled with FITC, and 40 µl of inoculum (~1×10^7 CFU/ml) were incubated with 25% (**A**) or 50% human blood (**B**). Data represents geometric mean of the fluorescence intensity (GMFI) ± SEM of an individual experiment performed in triplicate.

.
